# Hemoglobin induces inflammation through NF-kB signaling pathway and causes cell oxidative damage in grass carp (*Ctenopharyngodon idella*)

**DOI:** 10.3389/fimmu.2022.1044299

**Published:** 2022-11-23

**Authors:** Ying Tang, Shiyi Yang, Minshan Yao, Minxuan Yang, Lixiang Wei, Hong Chen, Junyan Lin, Yao Huang, Li Lin, Zhendong Qin

**Affiliations:** Guangdong Provincial Water Environment and Aquatic Products Security Engineering Technology Research Center, Guangzhou Key Laboratory of Aquatic Animal Diseases and Waterfowl Breeding, College of Animal Sciences and Technology, Zhongkai University of Agriculture and Engineering, Guangzhou, Guangdong, China

**Keywords:** Ctenopharyngodon idella, Hb, ROS, inflammation response, NF-κB, apoptosis

## Abstract

Hemolytic disease in grass carp (*C. idella*) leads to hemolysis *in vivo*, releasing damage-related molecular patterns (DAMPs) hemoglobin (Hb; which is rapidly oxidized to Hb-Fe^3+^ and Hb-Fe^4+^) and generating a high level of reactive oxygen species (ROS) that cause oxidative damage. However, the effect of cell-free Hb on tissue cells of grass carp has yet to be elucidated. In this study, western blotting (WB) and immunofluorescence analysis (IFA) results showed that PHZ-induced hemolysis caused Hb and iron accumulation, increased the production of ROS and resulted in apoptosis in head kidney and middle kidney of the grass carp. Quantitative real-time PCR (qRT-PCR), WB, and IFA revealed that PHZ-induced hemolysis significantly upregulated the expression of inflammation-related genes through activation of the NF-κB signaling pathway. To further explore the effect of Hb, three forms of Hb (Hb, MetHb, and FerrylHb) were prepared. The incubation with the different forms of Hb and heme markedly upregulated the expression of cytokine genes through NF-κB signaling pathway, which was further confirmed by a specific inhibitor (caffeic acid phenethyl ester, CAPE). Flow cytometry analysis data showed that the stimulation of different forms of Hb and heme increased the production of ROS, and resulted in apoptosis. In summary, our data suggest that the excess cell-free Hb released during hemolysis modulates the inflammatory response through activation of the NF-κB signaling pathway and causes cell oxidative damage and apoptosis.

## Introduction

The inflammatory reaction or inflammation refers to the body’s response to pathogen infection or mechanical damage and other physiological stimuli (1, 2). Inflammation is a defense state of the body and has a positive influence on the body. However, excessive inflammation can cause a certain degree of damage or even pathological changes to the body, as evidenced in atherosclerosis, diabetes, and rheumatoid arthritis (3–5). Inflammatory responses are usually accompanied by the release of pro-inflammatory cytokines (IL-1β, IL-6, TNF-α, etc.) and anti-inflammatory cytokines (IL-4, IL-10, IL-13, etc.), which are regulated through various signaling pathways (6). Inflammation-related signaling pathways, such as NF-κB, MAPK, and PI3K/Akt, have been widely studied (7, 8). NF-κB signal pathway activation occurs through extracellular signal stimulation of the IκB kinase (IKK) complex, phosphorylation of IκBα protein, and subsequent ubiquitination of IκBα protein. After degradation of IκBα, the p65-p50 heterodimer enters the nucleus and binds to the κB site on DNA to induce the production of cytokines (IL-1, IL-6, TNFα, etc.) (9–11). Previous research found that hemolytic oxidized hemoglobin (Hb) can activate the NF-κB pathway and induce endothelial cells to express pro-inflammatory genes such as E-selectin, ICAM-1, and VCAM (12).

Hb is a tetrameric globular protein composed of two α and two β polypeptide chains, each containing one heme (ferroprotoporphyrin IX) (13). The primary biological function of Hb is to transport oxygen. Under normal biological conditions, Hb is stored in red blood cells (RBCs), which contain numerous antioxidant enzymes to prevent Hb from being oxidized (14). During hemolysis *in vivo*, erythrocytes rupture and the protective effect of the antioxidant enzymes fails, leading to the release of cell-free Hb and the gradual production of ROS, including superoxide radicals (HO_2_·), H_2_O_2_, hydroxyl radicals (OH-), and superoxide anions (O_2_·-), ultimately culminating in disease or organism injury (15, 16). The typical characteristics of some human diseases are due to exposure of oxidized Hb and heme in the blood or tissues, including intraventricular hemorrhage (IVH), sickle cell disease (SCD), acute respiratory distress syndrome (ARDS), and hemolytic uremic syndrome (HUS) (17–20). In an acute lung injury model, Shaver et al. identified that Hb induced the NLRP3 inflammasome in mouse macrophages through a TLR4-dependent manner, increased the expression of IL-1β, and caused inflammation (17). In a model of IVH, oxidized Hb could upregulate inflammatory factors such as IL-1β, IL-6, IL-8, TNFα and so on, and also caused tissue apoptosis (18). In aquatic animals, Qin et al. established a grass carp hemolysis model through the injection of PHZ and showed that hemolysis upregulated various inflammatory factors in tissues (16); however, the specific mechanism of how grass carp Hb causes inflammation remains to be clarified.

Grass carp (*C. idella*) is an important freshwater farmed fish in China, with a wide range of culture methods and high economic value (21). According to the 2021 China Fishery Statistical Yearbook, the aquaculture output of grass carp was 5.571 million tons in 2020, ranking first in China. In recent years, the continuous expansion of culture density and scale has been accompanied by frequent occurrences of hemorrhagic diseases caused by various hemorrhagic pathogens such as *Aeromonas hydrophila* and grass carp reovirus (22, 23). These diseases can lead to hemolysis in the fish and release of excessive Hb into plasma or tissue, which may affect the health of the fish (16, 24).

In the current study, grass carp hemolysis was used as a model to explore the regulatory mechanism of Hb on inflammation through experiments *in vivo* and *in vitro*. Findings from the study provide a new idea for exploring the blood immune function of teleost fish.

## Materials and methods

### Experimental fish and treatments

Grass carp (150–250 g) were obtained from a farm located in Guangzhou City, Guangdong Province, China. The fish were cultured for two weeks in a tank with a water temperature of 27–28°C to adapt to the environmental conditions prior to the experiment.

### Phenylalanine (PHZ) induced hemolysis model

The hemolysis model was established by injection of PHZ. Briefly, experimental fish were randomly divided into two groups: one group were intraperitoneally injected with 40 mg/kg (body weight) of PHZ, and the second (control) group were injected with an equal volume of phosphate-buffered saline (PBS). The head and middle kidney were collected at 12, 24, 48 h, after injection of PHZ.

### Immunofluorescence analysis

IFA was performed according to the protocol described previously, with minor modification (16). The tissue sections were dewaxed with Safeclear II, rehydrated in graded ethanol (5 μM), and then heated with 10 mM sodium citrate for 15 min. The samples were blocked with 5% skimmed milk at room temperature (RT) for 2 h, then incubated with the primary anti-Hb and malondialdehyde (MDA) antibody (1:1000 in 2% skimmed milk) for 2 h at RT. After washing three times with PBST buffer, the FITC-labeled anti-rabbit IgG second antibody (1:10,000 in 2% skimmed milk) was added and incubated at 37°C for 30 min. Finally, the tissues were stained with DAPI for 5 min, then were observed and imaged under a fluorescence microscope (Olympus BX51, Japan).

### Perls iron staining

To determine the effect of PHZ-induced hemolysis on iron in tissues, the collected fish tissues were immobilized in 4% paraformaldehyde (Servicebio, Wuhan, China) for at least 12 h and then iron deposition was detected by Perls iron staining. The tissue sections were subsequently observed under a microscope (Axiostar Plus, Carl Zeiss, Germany) and imaged through a Canon camera PowerShotG6, 7.1 megapixel (Canon 219).

### Immunohistochemistry

IHC was performed to explore whether the PHZ-induced hemolysis impacted the expression of IκBα and affected the content of 4-HNE. Tissue sections were treated with 5% skimmed milk and blocked at RT for 2 h, then the primary IκBα and 4-HNE antibodies (1:1000 dilutions) were added, respectively. Subsequently, the polymerperoxidase-labeled secondary antibodies were incubated for 1 h at RT. Images of the tissues were obtained under an Olympus BX51 inverted microscope.

### Western blotting

The WB process was performed according to a previously described method (25). Protein samples were loaded and separated by sodium dodecyl sulfate–polyacrylamide gel electrophoresis (SDS-PAGE), then transferred to nitrocellulose membranes. Membranes were blocked in TBST containing 5% skimmed milk at RT for 3 h and then incubated with primary antibodies to Hb, IκBα, p50, and p65 (1:1,000 dilution) at 4°C overnight. After washing three times with TBST, the membranes were incubated with horseradish peroxidase (HRP)-linked goat anti-rabbit IgG secondary antibody (1:10,000 dilution) at RT for 30 min. Signals of the protein bands were detected using Clarity Western ECL Substrate (Solarbio Biology, China) and the membranes were imaged and analyzed using the ChemiDoc™ MP System (Bio-Rad).

### Total RNA extraction and cDNA synthesis

The methods for RNA extraction and cDNA synthesis were slightly modified as previously described (26, 27). Total RNA was extracted from the collected tissues using RNAiso Plus (Takara, Japan) and the quantity and quality of the RNA were determined using a NanoDrop 2000 spectrophotometer (Thermo Scientific) and an Agilent 2100 Bioanalyzer (Agilent Technologies, Santa Clara, CA, USA), respectively. Approximately 1 μg of total RNA was used to synthesize the first-strand cDNA using HIScript^®^ Q Select RT SuperMix for qPCR (Vazyme, Nanjing, China) following the manufacturer’s instructions and finally then stored at −20°C before use.

### Quantitative Real-Time PCR

To further explore the modulation effect of PHZ-induced hemolysis on tested genes, qRT-PCR was performed as previously described with slight modifications (28). The qRT-PCR amplification volume was 20 μL and included 4 μL diluted cDNA, 5 μL nuclease-free water, 0.5 μL of each gene-specific primer (10 mM, listed in **Table 1**), and 10 μL AceQ^®^ qPCR SYBR^®^ Green Master Mix (Vazyme, Nanjing, China). A qTOWER3 Real-Time PCR thermocycler (Analytik Jena AG, Germany) was used for the qRT-PCR according to following process: pre-incubation at 95°C for 30 s; 45 cycles of 95°C for 5 s, 55°C for 20 s, and 72°C for 20 s; and a final incubation at 4°C for 5 min. β-actin was used for cDNA normalization as an internal control gene. Statistical analysis used the 2^−△△CT^ method.

**Table 1:** List of primers used in the study.

Primer name	Sequences (5’-3’)
GcTNFa-RT-F	CGTATGGCGGGTGTGTGG
GcTNFa-RT-R	AAAGCCTGGTCCTGGTTC
GcIκBα-RT-F	CCTGCAAAAGAGGGTCGCTA
GcIκBα-RT-R	TTACATCTGCCCCAAGCTGG
GcIKKα-RT-F	GGCGTTCATGACACAAGCTG
GcIKKα-RT-R	AAAACAGCTTCTCGCCCGTA
Gcp50-RT-F	TCCCTGGAGAGGATGTACTCAAT
Gc p50-RT-R	TGTCAGTGTGATGTCTACCTTGG
GcTLR4-RT-F	GAAGTCCATCGCCTCCAACA
GcTLR4-RT-R	AAACCGGGACTGAGCCAATT
Gcβ-actin-RT-F	ACCCACACCGTGCCCATCTA
Gcβ-actin-RT-R	CCCATCTCCTGCTCGAAGTC
Gcp65-RT-F	TATTCCTGAAGCGAAGATCTGGG
Gcp65-RT-R	TTGGAGCTCTGTTGTCGTAGATG
GcCCL1-RT-F	CTTCAACCTGCCTTTGTCTCAAG
GcCCL1-RT-R	CGGAGCAAAAAGACAATCCTCTG
GcproIL-1β-RT-F	GCAGCCAAAGTGTTTACATGC
GcproIL-1β-RT-R	AAGCCCCGCACATGACATG
GcIL-1β-RT-F	ATGTGATCCAAGCACGTCGT
GcIL-1β-RT-R	ATCCGCTGCTTTTCACCAGA
GcIL-6-RT-F	AGAAATGGTATCTGATGGCA
GcIL-6-RT-R	GAAGGTCTGAGGAGAGGCGT
GcIL-8-RT-F	ATCTACGCTGTCGCTGCATT
GcIL-8-RT-R	AGCAGTAGGGTCCAGACAGA
GcIL-10-RT-F	CCCTTTGAGTTTGCCACCA
GcIL-10-RT-R	CAGCCATCATCCAATCCAC
GcCOX2-RT-F	GGAGCCCCCTACTCTCTCAA
GcCOX2-RT-R	ACCAGCTTCTTCAGTGAGGC
GcG-CSF-RT-F	AGGAAATCGGAGCCGTCTTG
GcG-CSFRT-R	CACTGTTGAGCTGCAGGGTA
GcNLRP3-RT-F	AGGAGTGTATCTGGACTGGCT
GcNLRP3-RT-R	AGGGGTTCAGTGAATGTGCT
GcTNFR1-RT-F	GCAGCAACCCATGGACAAAG
GcTNFR1-RT-R	AGGCGATGTGTTCTGCTGAA

### Erythrocyte separation and Hb preparation

Blood was collected from the caudal vein of fish in each group using heparinized syringes and was mixed with 0.7% buffered saline. The RBCs were isolated through Lymphocyte Separation Medium (Solarbio, Beijing, China), purified by centrifugation for 30 min at 4°C and 500 g, and collected after washing three times with 0.7% saline solution.

Hb (Fe^2+^), MetHb (Fe^3+^), and FerrylHb (Fe^4+^ = O) were prepared as described previously with minor modifications (29). Briefly, RBCs were thawed twice at RT, frozen at −80°C, and then centrifuged at 10000 g for 30 min. K_3_Fe (CN)_6_ was added and incubated at 25°C for 30 min to prepare MetHb. FerrylHb was prepared by incubating Hb with H_2_O_2_ at 37°C for 1 h. The appropriate proportion was determined by measuring the absorption spectrum at 500 to 700 nm. After oxidation, both MetHb and FerrylHb were dialyzed in PBS (three times for 3 h at 4°C) and stored at −80°C. The prepared Hb (Fe^2+^), MetHb (Fe^3+^), and FerrylHb (Fe^4+^) were identified by WB and silver staining kits.

### Cell culture and cell viability determination

L8824 cells were cultured in DMEM high glucose medium (biosharp, China) supplemented with 10% fetal bovine serum (FBS, GemCell, USA), 100 U/mL penicillin (Genom), and 100 µg/ml streptomycin (Genom). The cells were incubated at 28°C under a 5% CO_2_ humid atmosphere. In the treatment groups, L8824 cells in DMEM with 10% FBS were seeded at 1 × 10^6^/well in 12-well plates, and adherent cells were stimulated with 0.5, 1, and 2 mg/mL Hb, MetHb, and FerrylHb, and 10, 20, and 30 μM heme (Sigma, Cat# H3281). In the control group, the cells were incubated with an equal concentration of dimethyl sulfoxide (DMSO) as that used in the treatment groups. Cell viability was determined by the CCK-8 Cell Proliferation and Cytotoxicity Assay Kit (Solarbio Biology, China) after treatment with the above reagents for 12 and 24 h; the absorption spectrum was detected by a microplate reader at 450 nm.

### Apoptosis analysis

The effect of PHZ-induced hemolysis on apoptosis in tissues was investigated using tissue apoptosis methods that were slightly modified as described earlier (30). The tissue sections (5 μM) were stained using a TdT-mediated dUTP Notch End Labeling (TUNEL) one-step apoptosis detection kit (Guge Biology, Wuhan), followed by staining of cell nuclei with 4′, 6-diamino-2-phenyl indole (DAPI) (Servicebio, Wuhan, China) for 5 min at 25°C. Images were captured under a fluorescence microscope (Leica DMI8, Germany).

A flow cytometry method was used to determine whether incubation with Hb caused apoptosis. Briefly, treated cells were digested with trypsin and then 1× Annexin-binding buffer was added to make a 100 μL single cell suspension, which was incubated with 5 μL FITC-Annexin V and 1 μL propidium iodide (PI) working solution (100 μg/mL) for 15 min (Thermo Scientific, V13242). Subsequently, 400 μL of 1× Annexin-binding buffer was added and samples were analyzed by flow cytometry, measuring fluorescence emissions at 530 nm and 575 nm (BD Accuri C6 Plus).

### ROS determination

Total ROS levels in the collected tissues were measured with the fluorescent dye dihydroethidium (DHE). The frozen sections were incubated with 2 μM DHE for 30 min, then incubated with DAPI solution at 37°C for 5min, and observed by fluorescence microscopy (Leica DMI8, Germany). The signal intensity of the fluorescence was analyzed using Image J software.

To detect ROS in cells after stimulation with heme, Hb, MetHb, and FerrylHb, a flow cytometry method was utilized. After stimulation for 12 h, a final concentration of 50 μg/mL DCFH-DA probe was added into the cells and incubated at 28°C for 30min. A single cell suspension was then prepared by washing three times with PBS and was analyzed by flow cytometry, measuring the fluorescence emission at 530 nm.

### Statistical analysis

SPSS software (version 17.0) was used for statistical analysis. Data were expressed as mean ± standard deviation (SD). Statistical significance of the data was assessed using one-factor analysis of variance (ANOVA) and plotted using GraphPad Prism 7. After statistical analysis, the difference is shown as *P* < 0.05 (*) or *P* < 0.01 (**).

## Results

### PHZ-induced hemolysis caused tissue accumulation of Hb and injured the tissues

**Figure 1:**
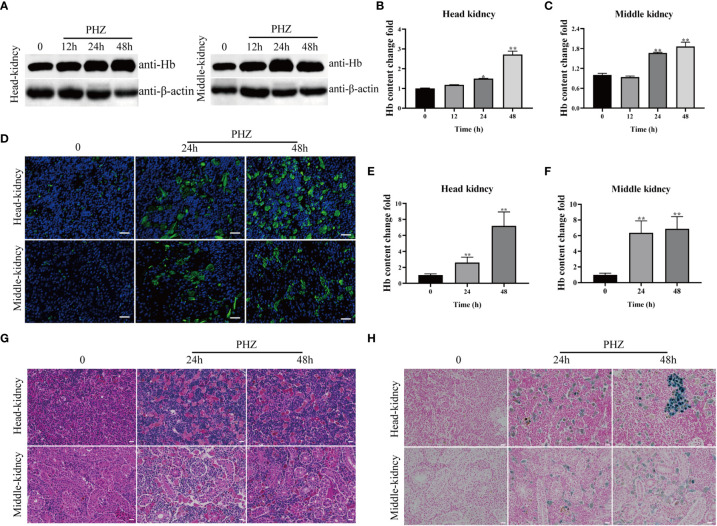
Hemolysis caused Hb accumulation and tissue injury. **(A–C)** WB was used to analyze the accumulation of Hb in head and middle kidney after injection of PHZ for 12 h, 24 h and 48 h and the corresponding grey ratios of Hb were analysed by image J. **(D)** The accumulation of Hb in head and middle kidney were determined by IFA assay after injection of PHZ for 24 h and 48 h, the scale bar represents 20 μm. **(E, F)** The content of Hb in head **(E)** and middle kidney **(F)** after injection of PHZ for 24 h and 48 h. **(G)** The head and middle kidney were stained with hematoxylin-eosin staining at 24 h and 48 h after injection of PHZ, the scale bar represents 20 μm. **(H)** Prussian blue stained head and middle kidney at 24 h, and 48 h after PHZ injection, the scale bar represents 20 μm.*P<0.05; **P< 0.01 (Student’s t-test). The data were the mean values of three independent experiments, expressed as mean ± SEM(*P < 0.05, **P < 0.01.).

In this experiment, a hemolysis model was constructed by injection of PHZ into grass carp, and samples were collected at 12, 24, and 48 h. WB and IFA were used to detect cellular Hb accumulation in the head kidney and middle kidney of the grass carp. PHZ-induced hemolysis significantly increased the level of Hb at 24 and 48 h in both the head and middle kidney of the fish (**Figures 1A–F**). To explore the damage caused to the tissues by hemolysis, HE staining was performed and revealed that PHZ-induced hemolysis markedly increased the accumulation of Hb and induced obvious cellular damage in both the head kidney and middle kidney at 24 and 48 h compared with the control group (**Figure 1G**). In addition, macrophage phagocytosis of the deposited Hb was observed in the head kidney and was particularly evident at 48 h, while in the middle kidney, tubular vacuolization and interstitial gap enlargement were present (**Figure 1G**). A Perls iron staining assay was performed to further test whether the Hb accumulated in the tissues affected iron deposition. The results of this assay revealed that large amounts of iron ions were deposited in the head and middle kidneys at both 24 and 48 h (**Figure 1H**).

### PHZ-induced hemolysis increased ROS levels and caused tissue oxidative damage

**Figure 2:**
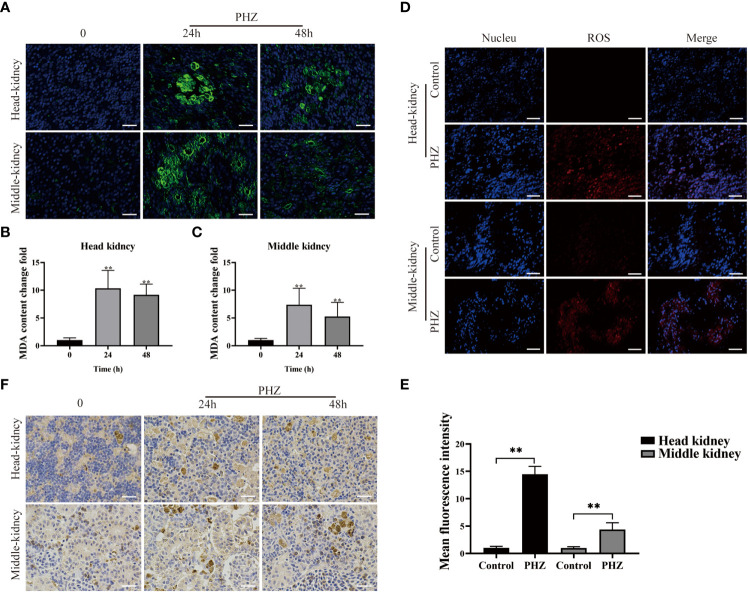
PHZ-induced hemolysis increased the generation of ROS and caused tissue oxidative damage. **(A) **The degree of MDA immune response was detected by green display after injection of PHZ for 24 h and 48 (h) Scale bar, 20 μm. **(B, C)** The content of MDA levels in head kidney **(B)** and middle kidney **(C)** at 24 and 48 h after injection of PHZ. **(D, E)** The generation of ROS in head kidney and middle kidney **(E)** were measured by fluorescence probe DHE after injection of PHZ. Scale bar, 20 μm. **(F)** The IHC was used to analysis the content of 4-HNE in head and middle kidney after injection of PHZ for 24 and 48 (h) Scale means 20 μm. *P < 0.05, **P < 0.01. The data were the mean values of three independent experiments, expressed as mean ± SEM.

To investigate the effect of PHZ-induced hemolysis on tissue oxidative stress, ROS levels in the head kidney and mid kidney of the grass carp were measured using a DHE fluorescent probe. Production of ROS significantly increased in the head and middle kidneys of PHZ-treated fish compared with the control group (**Figures 2D, E**). The extent of oxidative damage caused to the tissue by hemolysis was evaluated by measuring the content of the oxidative damage markers MDA and 4-HNE. PHZ-induced hemolysis significantly increased MDA content at 24 and 48 h in the head kidney, and similar results were observed in the middle kidney (**Figures 2A–C**). In the detection of 4-HNE, obvious brown precipitates were observed in the head and middle kidneys 24 and 48 h after injection of PHZ compared with the control group (**Figure 2F**).

### Hemolysis modulated inflammation responses *via* the NF-κB signaling pathway

**Figure 3:**
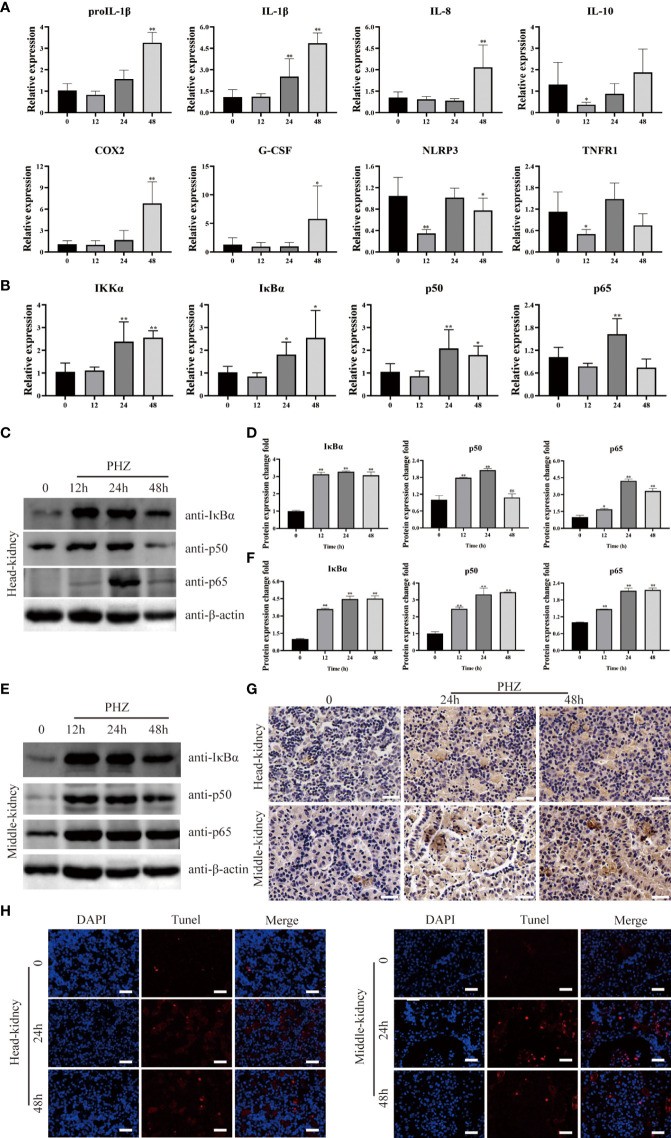
Hemolysis can regulate inflammation through NF-κB signaling pathway. **(A, B)** The qRT-PCR was used to detect the mRNA expression levels at 12 h, 24 h and 48 h after PHZ injection in head kidney, including ProIL-1β, IL-1β, IL-8, IL-10, COX2, G-CSF, NLRP3, TNFR1 **(A)** and IKKα, IκBα, P50, P65 **(B)**. **(C–F)** The protein expression levels of IκBα, P50 and P65 in the head kidney **(C, D)** and middle kidney **(E, F)** after PHZ injection 12 h, 24 h, and 48 h were detected by WB, and the gray scale analysis was performed by image J **(G)** The IHC was used to analysis the expression of IκBα in head and middle kidneys after injection of PHZ 24 and 48 h. Scale means 20μm. **(H)** TUNEL staining was used to detect the apoptosis of head and middle kidney (red) after injection of PHZ 48 h. The scale bar was 20 μm. The data were the mean values of three independent experiments, expressed as mean ± SEM ( *P < 0.05, **P < 0.01.).

To investigate the relationship between hemolysis and inflammation, the expression of inflammation-related genes was determined in the head kidney by qRT-PCR at different time points after PHZ injection. Hemolysis induced by PHZ markedly elevated the expression of proIL-1β and IL-1β at 24 and 48 h (**Figure 3A**). IL-8, COX2, and G-CSF mRNA transcript levels were significantly upregulated by PHZ-induced hemolysis at 48 h, but were not significantly different from levels in the control group at 12 and 24 h. In contrast, the mRNA expression of IL-10 and TNFR1 was significantly downregulated at 12 h after PHZ injection, without obvious changes at 24 and 48 h. In addition, the expression level of NLRP3 was significantly decreased at 12 and 48 h after PHZ injection compared with the control group (**Figure 3A**). Next, to explore whether inflammation due to hemolysis was associated with the NF-κB pathway, NF-κB pathway-related genes, including IκBα, IKKα, p50, and p65, were examined by qRT-PCR. PHZ-induced hemolysis significantly upregulated the expression of IκBα, IKKα, and p50 at 24 and 48 h, and at 24 h for P65 (**Figure 3B**). WB revealed that the protein expression levels of IκBα, p50, and p65 were significantly increased in both the head kidney and middle kidney in fish injected with PHZ compared with the control group, which was also verified by grayscale analysis (**Figures 3C–F**). Furthermore, IHC analysis indicated that the level of IκBα in the PHZ-treated head kidney was markedly increased compared with that of the control group, and similar results were found in the middle kidney (**Figure 3G**). To examine the relationship between hemolysis-induced inflammation and apoptosis, TUNEL staining was used to detect apoptosis in the head and middle kidney cells. The TUNEL signal in the middle kidney was clearly increased at 24 and 48 h after treatment with PHZ, and there was a similar trend in the head kidney (**Figure 3H**).

### Incubation with Hb caused cell damage

**Figure 4:**
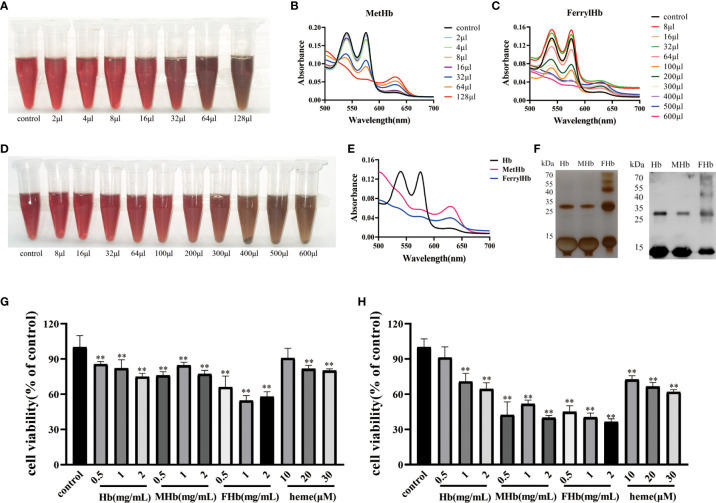
The stimulation of Hb caused cell damage. **(A, B)** The MetHb was prepared by incubating 30 min with incremental K_3_Fe(CN)_6_ at 25°C. The color changes were recorded and spectroscopic carried out to analyze the change of spectrum curve. **(C, D)** The FerrylHb was prepared by adding 3% H_2_O_2_ and incubating at 37°C for 1 h. The color change was recorded and spectroscopic carried out to analyze the change of spectrum curve. **(E)** The changes of the spectroscopic of Hb, MetHb and FerrylHb were compared. **(F)** Hb, MetHb and FerrylHb were silver stained by SDS-PAGE. **(G, H)** The survival rate of L8824 cells under stimulation of different concentrations of Hb, MetHb and FerrylHb for 12h **(G)** and 24h **(H)**. The data were the mean values of three independent experiments, expressed as mean ± SEM (**P < 0.01.).

To investigate which valence of Hb was associated with the triggering of inflammation and apoptosis in fish cells, Hb, MetHb, and FerrylHb were prepared and analyzed. MetHb was successfully prepared by adding 128 μL K_3_Fe (CN) _6_ to 500 μL Hb (**Figures 4A, B**). FerrylHb was prepared by adding incremental amounts of 3% H_2_O_2_ to Hb as confirmed by spectral curve analysis (**Figures 4C, D**). To verify that the three prepared compounds were different, the samples were examined using spectral curves, silver staining, and WB, and the results revealed that Hb and MetHb existed as monomer subunits of 16 kDa, while covalently cross-linked Hb polymers were detected in FerrylHb preparations (**Figures 4E, F**). After obtaining preparations with different valences of Hb, the toxic effect of the preparations was tested on L8824 cells using a CCK-8 kit. Incubation with Hb, MetHb, and FerrylHb at a final concentration of 2 mg/mL for 12 h significantly attenuated the viability of L8824 cells, and the survival rate of the FerrylHb-treated cells even decreased to 60% (**Figure 4G**). Meanwhile, cells incubated with heme showed no obvious change at 10 μM, but cell survival was decreased at 20 and 30 μM (**Figure 4G**). After 24 h of incubation, all tested concentrations and preparations of Hb exhibited a significant toxic effect on the viability of L8824 cells, especially FerrylHb and MetHb, with the survival rate decreasing to 40–50% (**Figure 4H**).

### Hb and heme promote the expression of cytokines *via* the NF-κB signaling pathway in L8824 cells

**Figure 5:**
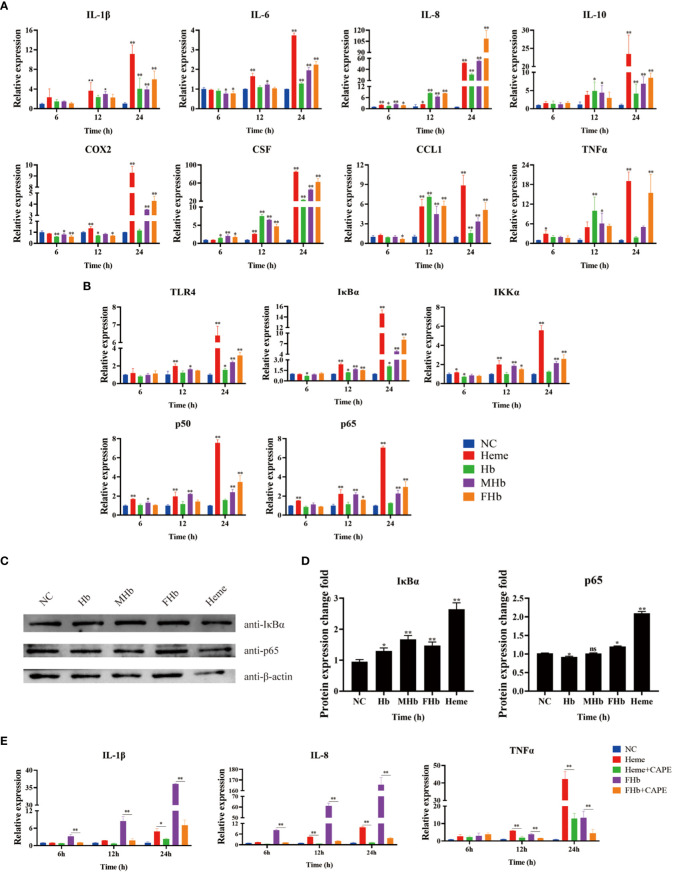
Hb, MHb and FHb modulated inflammation through NF-κB signaling pathway. **(A, B)** The qRT-PCR was used to detected the differential expression levels of mRNA including IL-1β, IL-6, IL-8, IL-10, COX2, CSF, CCL1, TNFα **(A)** and TLR4, IKKα, IκBα, P50,P65 **(B)** in L8824 cells infected with heme and different Hb for 6, 12 and 24 h respectively. **(C, D)** After L8824 cells were infected with heme and different Hb for 12 h, the protein expression levels of IκBα and p65 were detected by WB, and gray scale analysis was performed by image J **(E)**. The expression level of mRNA of IL-1β, IL-8 and TNFα were detected after L8824 cells were infected with FerrylHb and Heme with caffeic acid phenethyl ester (CAPE, 25μM) for 6, 12 and 24 h, respectively. The data were the mean values of three independent experiments, expressed as mean ± SEM (*P < 0.05, **P < 0.01.).

To explore the connection between different valences of Hb and inflammation, the expression of various cytokines was detected in L8824 cells after incubation with heme or different valences of Hb for 6, 12, and 24 h. qRT-PCR analysis demonstrated that the expression of IL-1β and IL-10 was not significantly changed after treatment for 6 h, while the expression of IL-8, and CSF was significantly elevated in almost all treatment groups (**Figure 5A**). After treatment for 12 h, IL-1β and IL-6 expression increased in the heme and MetHb groups, IL-8, CSF, and CCL1 expression markedly increased in all treatment groups, and IL-10 and TNFα expression showed a significant increase after Hb and MetHb treatment. The expression level of almost all detected cytokines was markedly improved after stimulation with various Hb preparations or heme for 24 h, especially in the heme- and FerrylHb-treated cells (**Figure 5A**). To investigate whether the inflammation caused by Hb and heme was regulated by the TLR4-NF-κB pathway, the expression of TLR4-NF-κB pathway-related genes was examined in the treated cells by qRT-PCR and WB. Firstly, qRT-PCR revealed that the mRNA transcript level of TLR4 was markedly increased in heme-treated cells at 12 h, and significantly upregulated in all treated groups at 24 h (**Figure 5B**). The expression of IκBα and IKKα were increased at 12 h and 24 h in almost all treated groups. Incubation with heme and MetHb markedly improved the expression of p50 and p65 at all almost tested time points, while stimulation with FerrylHb increased the mRNA transcript level of p50 at 24 h, and at 12 and 24 h for p65 (**Figure 5B**). Subsequently, WB revealed that the expression of IκBα was considerably higher in the treatment groups compared with the control group, especially in the heme-treated group (**Figures 5C, D**). Expression of p65 protein was significantly upregulated in FerrylHb and heme groups (**Figures 5C, D**).

The regulation relationship between inflammation and the NF-κB signaling pathway was further explored using the NF-κB inhibitor caffeic acid phenethyl ester (CAPE, 25 μM). The qRT-PCR data suggested that supplementation with CAPE significantly decreased the expression of IL-8 and TNFα (which were upregulated by heme) at 12 and 24 h, and attenuated the expression of IL-1β at 24 h (**Figure 5E**). Co-incubation of FerrylHb and CAPE markedly decreased the expression of IL-1β, IL-8, and TNFα at almost all detected time points compared with FerrylHb-treated cells, except TNFα at 6 h (**Figure 5E**).

### Incubation with Hb caused ROS and apoptosis

**Figure 6:**
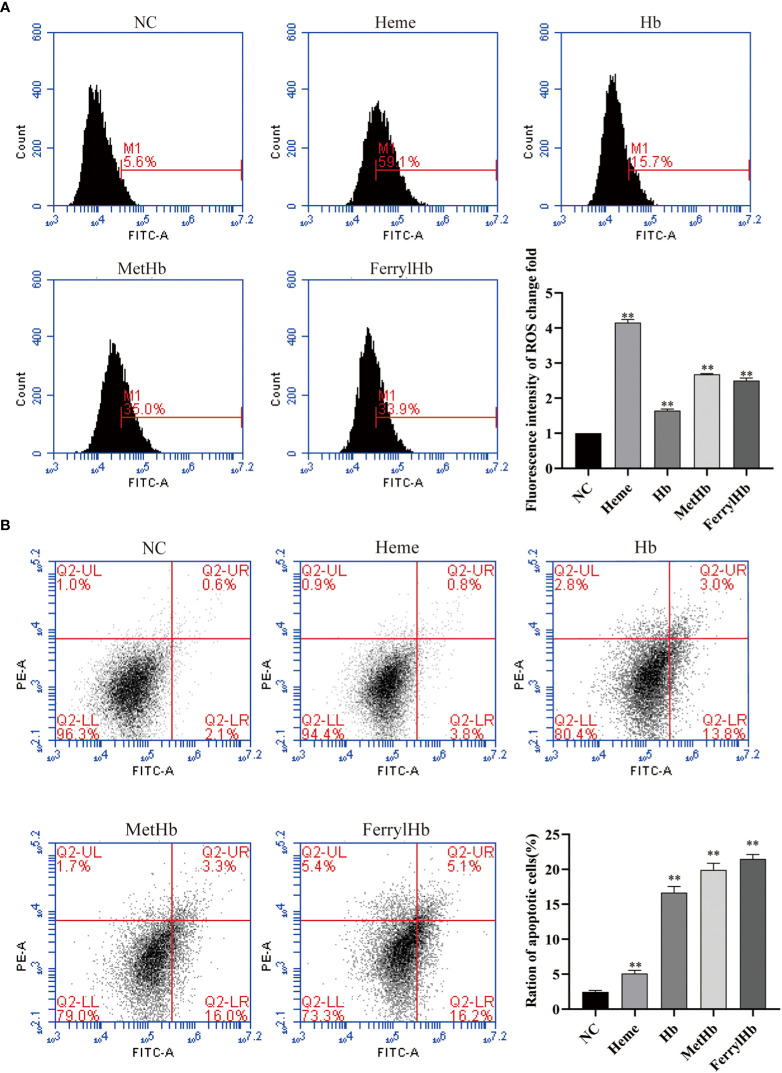
Different valence of Hb increased the production of ROS and apoptosis of L8824 cells. **(A)** Detection of ROS content of heme and different Hb infected L8824 cells after 12h by flow cytometry using DCFH-DA probe **(B)** Effect of heme and different Hb on apoptosis after incubation of L8824 cells for 12 h using Annexin V and PI dye. Data are the mean of three independent experiments and are expressed as mean ± SEM (**P < 0.01.).

To investigate the effect of different valences of Hb on ROS production, the DCFH-DA probe was used to label the ROS content of L8824 cells. Flow cytometry analysis of the labeled cells revealed that stimulation with heme, Hb, MetHb, and FerrylHb markedly increased the production of ROS in L8824 cells, with the largest increase observed in the heme-treated cells (**Figure 6A**). Finally, to determine whether the oxidative stress induced by different valences of Hb caused apoptosis, L8824 cells were incubated with heme or the different preparations of Hb and apoptosis was detected by flow cytometry following annexin V and PI staining. After 12 h, the apoptosis rate in heme-, Hb-, MetHb-, and FerrylHb-treated cells was markedly higher compared with that in control cells (**Figure 6B**).

## Discussion

Hb is stored in erythrocytes under normal conditions, and these cells contain a large number of antioxidant enzymes that prevent the oxidation of Hb (31–33). The occurrence of disease or tissue damage in an organism may lead to intravascular hemolysis, resulting in the release of free Hb, which is oxidized to produce Hb-Fe^3+^ and Hb-Fe^4+^, and the release of heme (34, 35). In mammalian models, high levels of oxidized Hb or heme were found to accompany the development of numerous diseases (36, 37). In the process of aquaculture, various hemorrhagic pathogens can cause hemolysis in fish, releasing free Hb and heme, threatening fish health, and limiting the development of aquaculture (38). In the present study, a sterile hemolysis grass carp model was constructed by injection of PHZ, and the Hb content in the head kidney and middle kidney were examined by WB, IFA, and HE staining, which confirmed that Hb accumulated in the head kidney and middle kidney under hemolysis. Meanwhile, Prussian blue staining assay revealed that PHZ-induced hemolysis led to iron ion deposition, which is similar to what happens in SCD as reported by Nader (19).

Upon hemolysis, cell-free Hb-Fe^2+^ is automatically oxidized to Hb-Fe^3+^ and generates superoxide anion radicals (O_2_·-), which in turn are converted to H_2_O_2_. In the presence of H_2_O_2_, a peroxidase (POX) catalytic cycle is initiated between Hb-Fe^3+^ and Hb-Fe^4+^, with simultaneous generation of superoxide radicals (HO_2_·) (15). In addition, the superoxide anion (O_2_·-) can form highly reactive hydroxyl radicals (OH-) through the Fenton reaction (39). A cascade of oxidative reactions leads to increased levels of ROS, and these high levels of ROS can trigger inflammation and oxidative tissue damage (40, 41). In the current study, the total ROS level was measured in the tissues of the sterile hemolysis model of grass carp using the oxidative fluorescent dye DHE, and this confirmed that hemolysis led to an increase in the production of ROS in the head kidney and mid kidney. Subsequently, the levels of the oxidative stress markers 4-HNE and MDA were measured by IHC and IFA, respectively. This illustrated that excess Hb caused by hemolysis led to oxidative damage in both the head kidney and middle kidney of grass carp. High levels of ROS are known to induce apoptosis (42). Recently, in an IPEC-J2 cells model, ROS accumulation was shown to activate apoptotic pathways, culminating in apoptosis (43). Furthermore, Somanathapura found that MetHb promotes platelet apoptosis through mitochondrial ROS-mediated activation of JNK and p38 MAP kinases (44). In the present study, the effect of hemolysis on apoptosis in the head kidney and mid kidney of grass carp was examined using the TUNEL staining method, and the resulting data clarified that hemolysis increased apoptosis in head and mid kidney cells, which is congruent with previous studies.

Previous studies have also indicated that free Hb in mammalian models leads to increases in the secretion of pro-inflammatory and chemotactic effector molecules IL-1β, TNFα, MCP-1, IL-8, and IL-6 (18). Consistently, Nyakundi et al. constructed a mouse model of aseptic hemolysis by injection of PHZ and found that oxidized Hb upregulated the expression of the pro-inflammatory cytokine IL-1β (29). In the present study, the mRNA expression of pro-inflammatory cytokines IL-1β, IL-8, COX2, and G-CSF were significantly increased at 48 h in the PHZ-treated group compared with the control group. Ciara et al. highlighted that cell-free Hb could activate the NLRP3 inflammasome, resulting in increased expression of IL-1β in a mouse model (17). Meanwhile, Lu et al. suggested that stimulation of Hb upregulates the expression of inflammatory cytokines including IL-1β and IL-8 through the NF-κB pathway (38). Therefore, we examined NF-κB-related gene expression levels in the head kidney of the grass carp hemolysis model and found that the PHZ-induced hemolysis significantly upregulated the expression of IκBα, IKKα, p50, and p65 mRNA at 24 and 48 h. Furthermore, WB and IFA revealed that PHZ-induced hemolysis differentially upregulated the protein expression level of IκBα, p50, and p65 at 12, 24, and 48 h. The above data implied that modulation of the inflammatory cytokine response by hemolysis was associated with activation of the NF-κB signaling pathway.

During hemolysis, owing to the Fenton reaction, Hb-Fe^2+^ can be oxidized to Hb-Fe^3+^ and Hb-Fe^4+^, and usually releases free heme and iron (15, 45). In the present study, the Prussian blue staining assay confirmed that iron was significantly accumulated in the PHZ-treated tissues. According to previous studies, intravascular hemolysis subsequently released Hb and heme, resulting in activation of the inflammatory response (46, 47). To investigate the specific mechanism of inflammation caused by different valences of Hb, we prepared Hb-Fe^2+^, Hb-Fe^3+^, and Hb-Fe^4+^. CCK-8 assay results revealed that incubation of L8824 cells with Hb-Fe^2+^, Hb-Fe^3+^, Hb-Fe^4+^, and heme markedly decreased the cell survival rate, especially Hb-Fe^4+^. Subsequently, the mRNA expression levels of cytokines were quantified in L8824 cells after incubation with the different Hb preparations and heme, and this revealed that the expression of pro-inflammatory cytokines was almost significantly upregulated in heme- and Hb-Fe^4+^-treated cells, especially at 24 h. A similar result was reported by Nyakundi et al. in mammalian models (29). Additionally, the expression levels of TLR4 and NF-κB-related genes were measured in L8824 cells after incubation with different Hb preparations and heme, and both qRT-PCR and WB indicated that stimulation with different forms of Hb and heme activated the NF-κB-associated pathway. Subsequently, the NF-κB inhibitor CAPE was used to further explore whether the inflammation activated by heme and Hb-Fe^4+^ occurred through the NF-κB pathway, and the data revealed that the co-incubation with CAPE significantly reduced inflammation compared with heme- and Hb-Fe^4+^-treated cells.

In conclusion, we utilized a sterile hemolysis model in grass carp by injection of PHZ and demonstrated that the PHZ-induced hemolysis led to Hb and iron accumulation, increased the production ROS, and caused oxidative damage and apoptosis. PHZ-induced hemolysis was also confirmed to upregulate the expression of inflammation, predominantly through NF-κB. Subsequently, we confirmed that three forms of Hb had a toxic effect on L8824 cells and activated the inflammatory response through the NF-κB pathway, ultimately increasing the production of ROS and apoptosis.

## Data availability statement

The raw data supporting the conclusions of this article will be made available by the authors, without undue reservation.

## Ethics statement

The animal study was reviewed and approved by the Animal Ethics Committee of Zhongkai University of Agriculture and Engineering.

## Author contributions

YT and SY performed experiments, analyzed the data, and wrote the manuscript. MXY, LX, HC, JL, YH performed the experiments. LL and ZQ conceived ideas, analyzed the data, oversaw the research, and wrote the manuscript. All authors contributed to the article and approved the submitted version.

## Funding

This work was jointly supported by the National Natural Science Foundation of China (31902409, 31872606, 31572657, U1701233); Key Project of Department of Education of Guangdong Province (2020ZDZX1026).

## Conflict of interest

The authors declare that the research was conducted in the absence of any commercial or financial relationships that could be construed as a potential conflict of interest.

## Publisher’s note

All claims expressed in this article are solely those of the authors and do not necessarily represent those of their affiliated organizations, or those of the publisher, the editors and the reviewers. Any product that may be evaluated in this article, or claim that may be made by its manufacturer, is not guaranteed or endorsed by the publisher.
